# Supplementary figures and images for: β-catenin mediates growth defects induced by centrosome loss in a subset of APC mutant colorectal cancer independently of p53
Source: PLoS One. 2024 Feb 7;19(2):e0295030. doi: 10.1371/journal.pone.0295030 (PMC10849215; doi:10.1371/journal.pone.0295030)

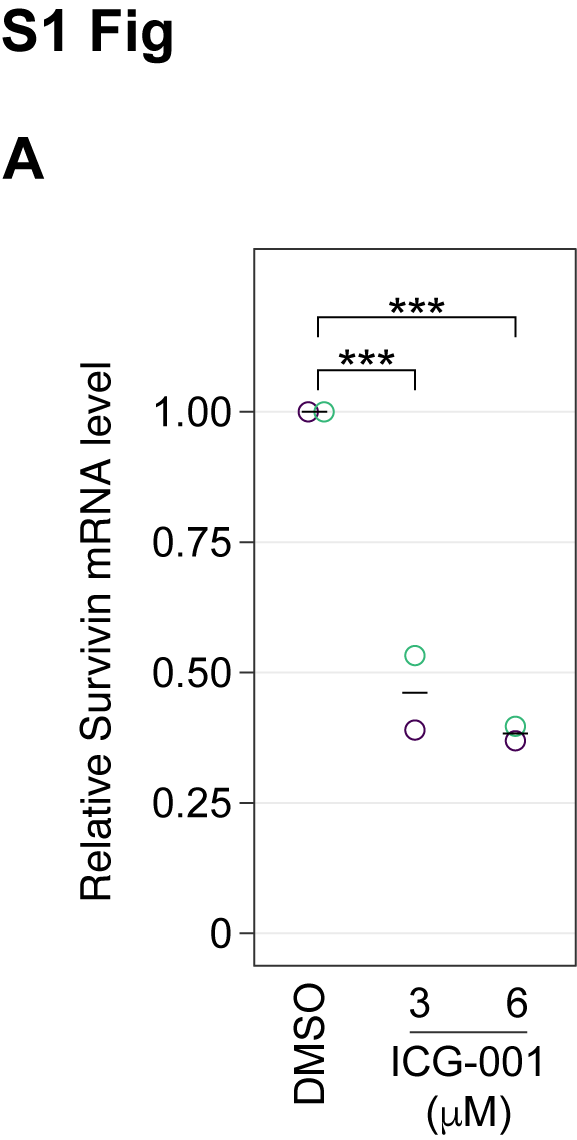

Supplement: S1 Fig — A) MDA-MB-231 cells were treated with DMSO or 3 or 6 μM ICG-001 for 72 h before collecting cells for mRNA extraction. qPCR for surviving was performed on the subsequent cDNA using RPLP0 as a control. Relative survivin mRNA was normalized using RPLP0 and compared to levels in DMSO-treated cells. (n = 2, ***P<0.001, One-way ANOVA with Dunnett post-hoc using DMSO as control). (TIF) [file pone.0295030.s001.tif]

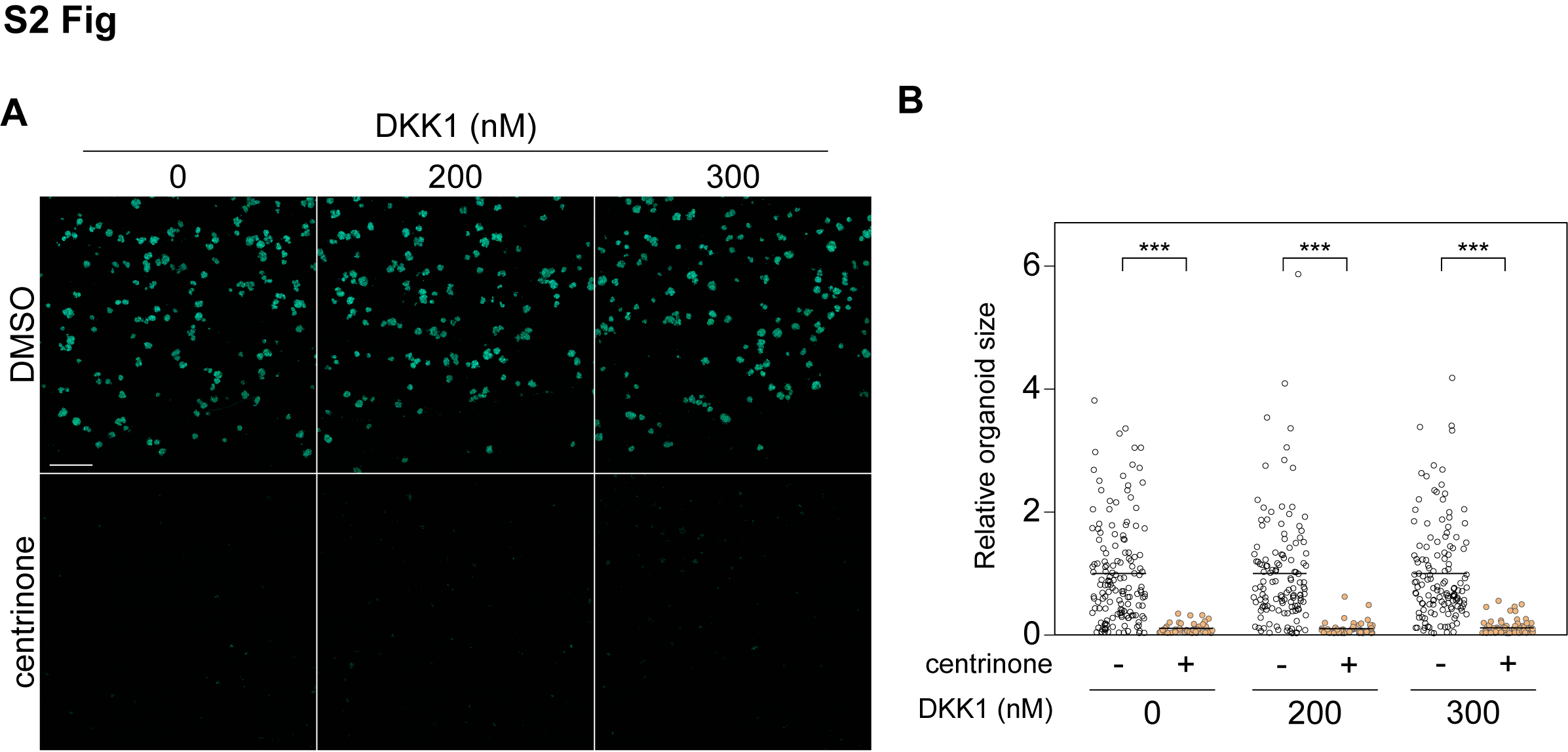

Supplement: S2 Fig — A) CSC-406 cancer HCOs were grown from an equal number of single adult stem cells in the presence of DMSO or the indicated concentrations of DKK1 and DMSO or 0.5 μM centrinone B for 8 days. Organoids were then fixed, stained with DAPI to label nuclei and phalloidin to label actin, and imaged. B) The areas of individual organoids from (A) were quantified in the merged maximum intensity projection images and the relative values are presented in the graph, every dot represents an organoid. (n = 1, ***P<0.001, Mann-Whitney U test). (TIF) [file pone.0295030.s002.tif]
