# Supplementary material for: β-catenin mediates growth defects induced by centrosome loss in a subset of APC mutant colorectal cancer independently of p53
Source: PLoS One. 2024 Feb 7;19(2):e0295030. doi: 10.1371/journal.pone.0295030 (PMC10849215; doi:10.1371/journal.pone.0295030)
Supplement: S1 Raw images — Boxed regions indicate cropped area used for Figure preparation. (PDF) [file pone.0295030.s005.pdf]

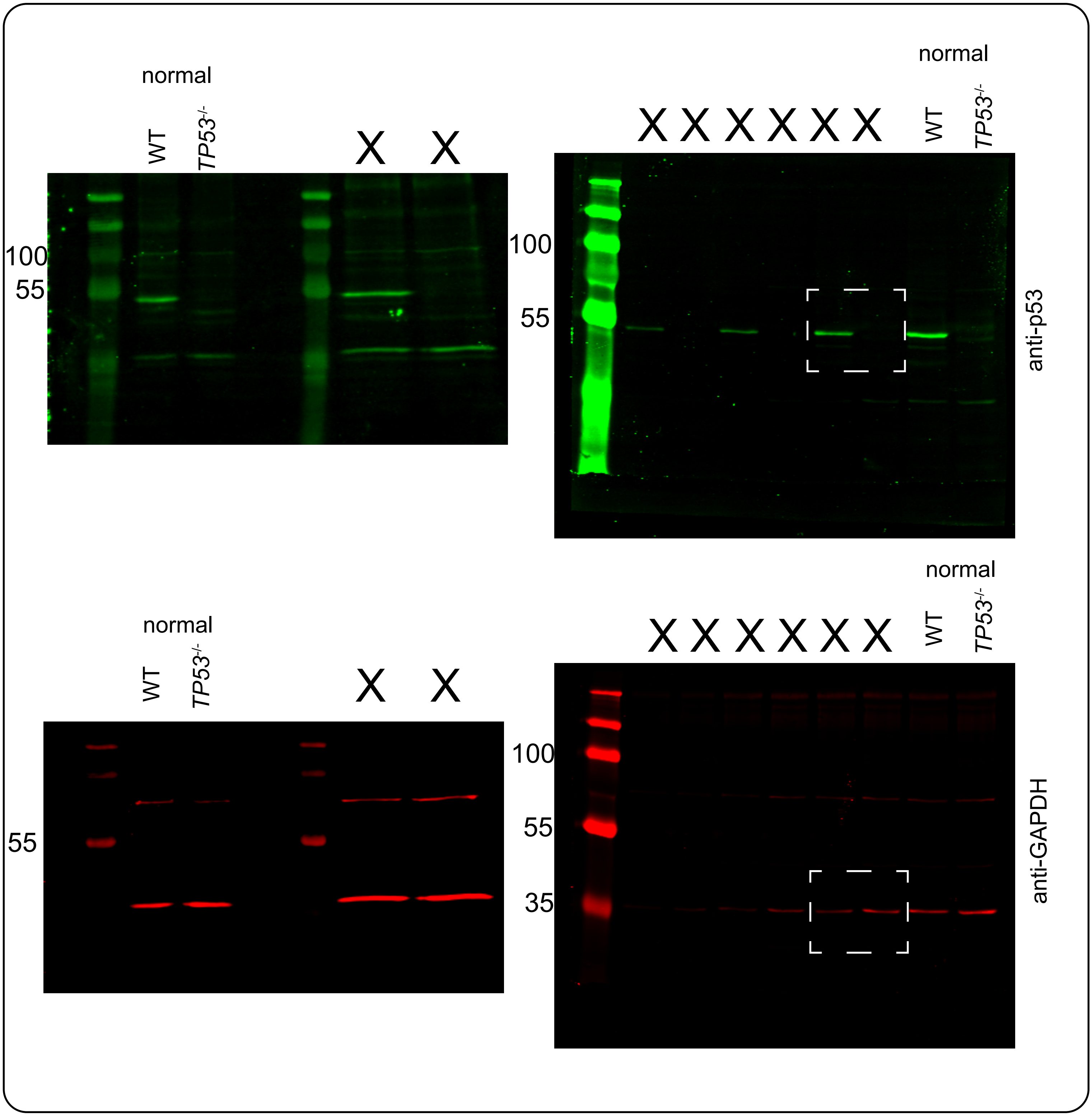

Used for Panel 1E. Boxed regions used for figure.

All Western blot images obtained using LI-COR Odyssey CLx Imager.

Page 1

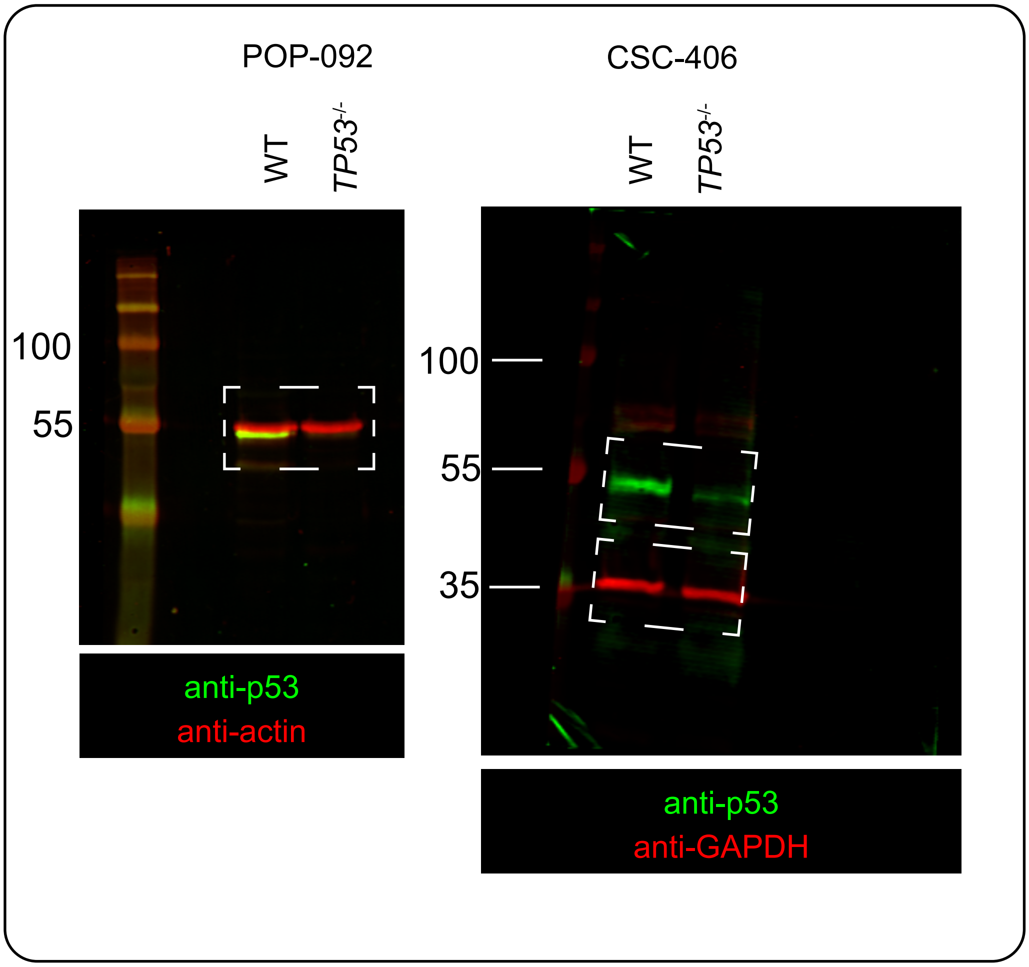

Data on Page2 and 3 used for Panel 2E.
Boxed regions used for figure.

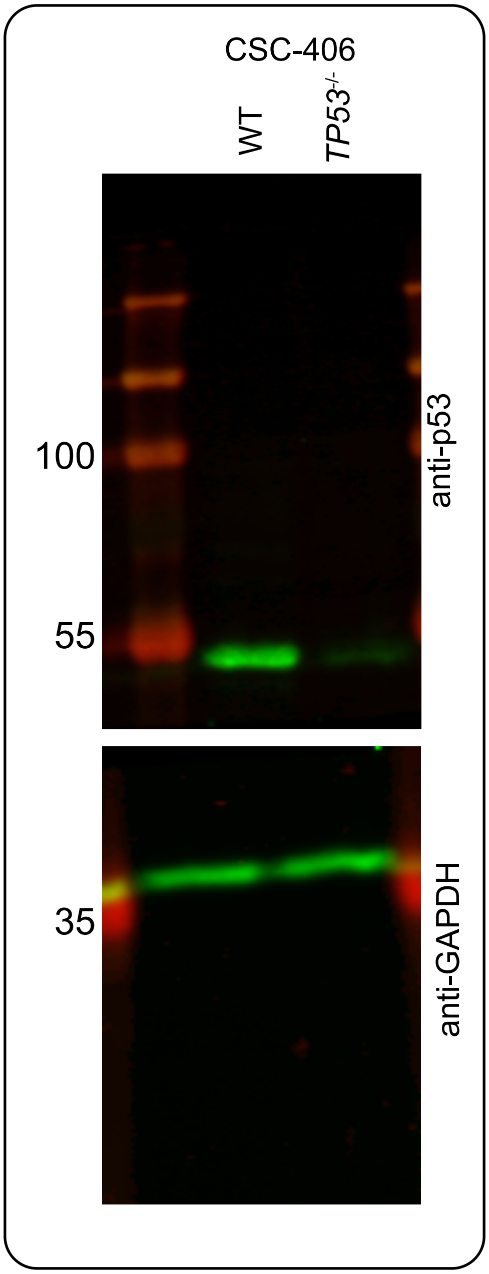
CSC-406
WT
TP53-/-
100
55
anti-p53
35
anti-GAPDH

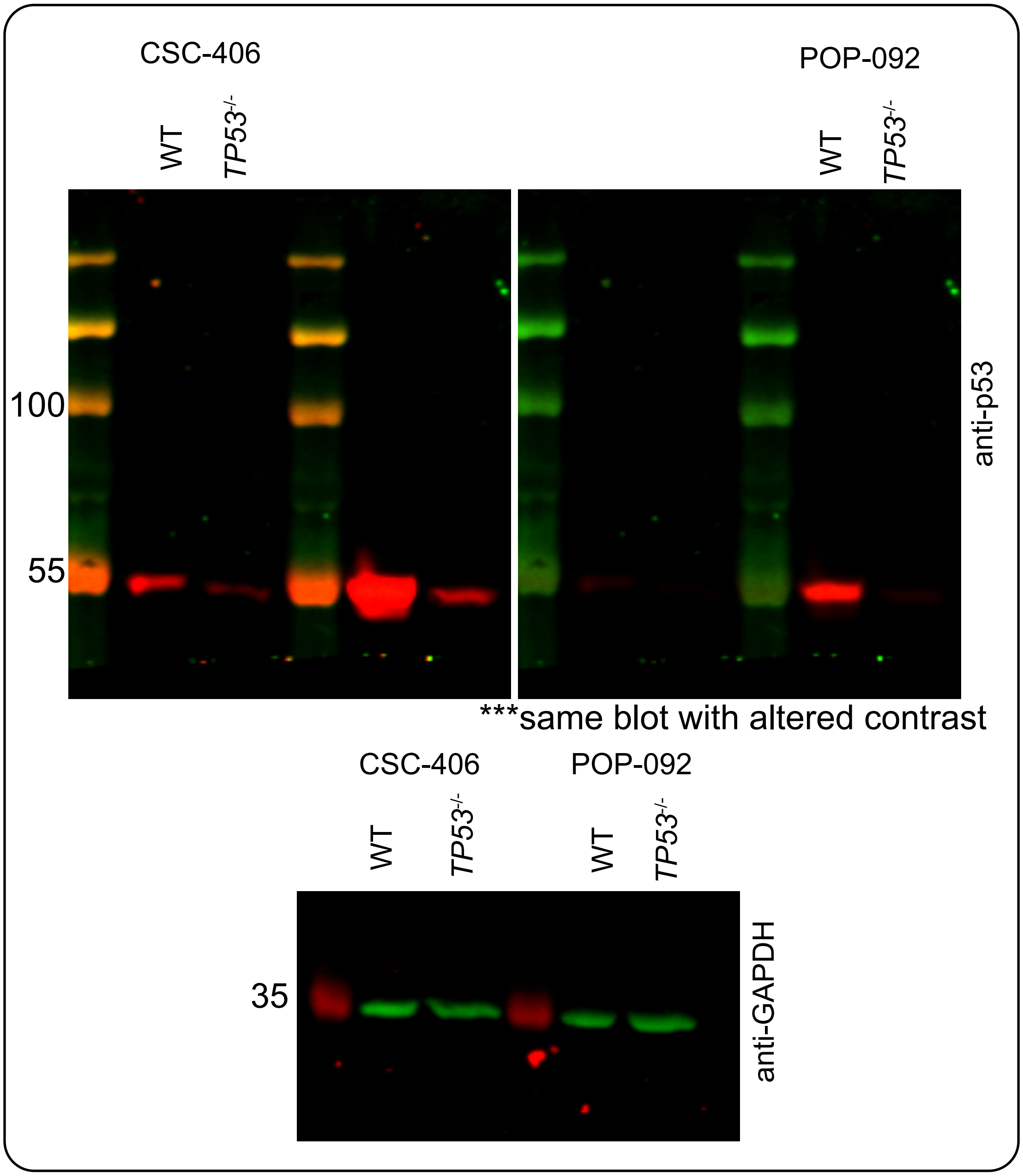
CSC-406
POP-092
WT
TP53-/-
WT
TP53-/-
100
55
anti-p53
***same blot with altered contrast
CSC-406
POP-092
WT
TP53-/-
WT
TP53-/-
35
anti-GAPDH

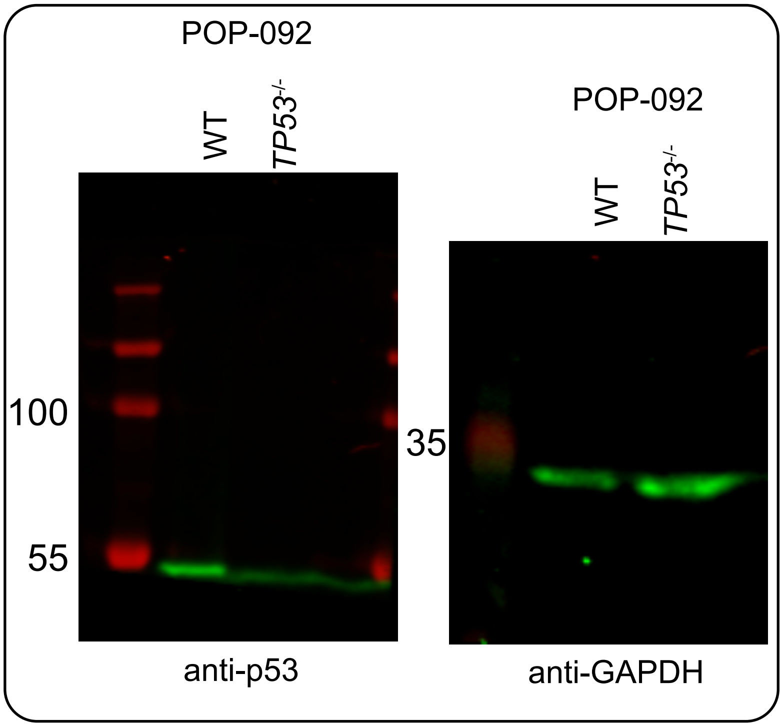
POP-092
WT
TP53-/-
100
55
anti-p53
POP-092
WT
TP53-/-
35
anti-GAPDH

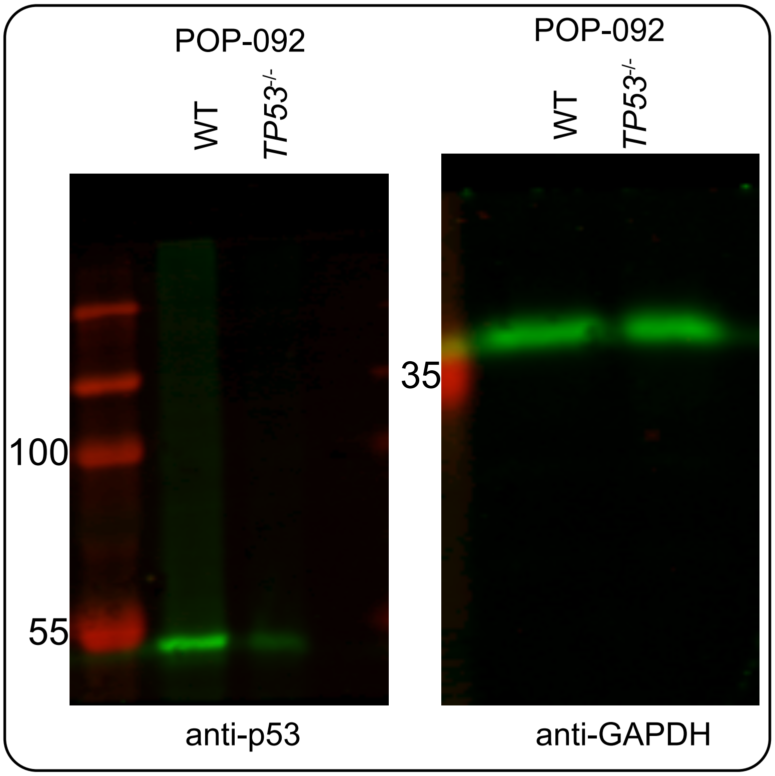
POP-092
WT
TP53-/-
100
55
anti-p53
POP-092
WT
TP53-/-
35
anti-GAPDH

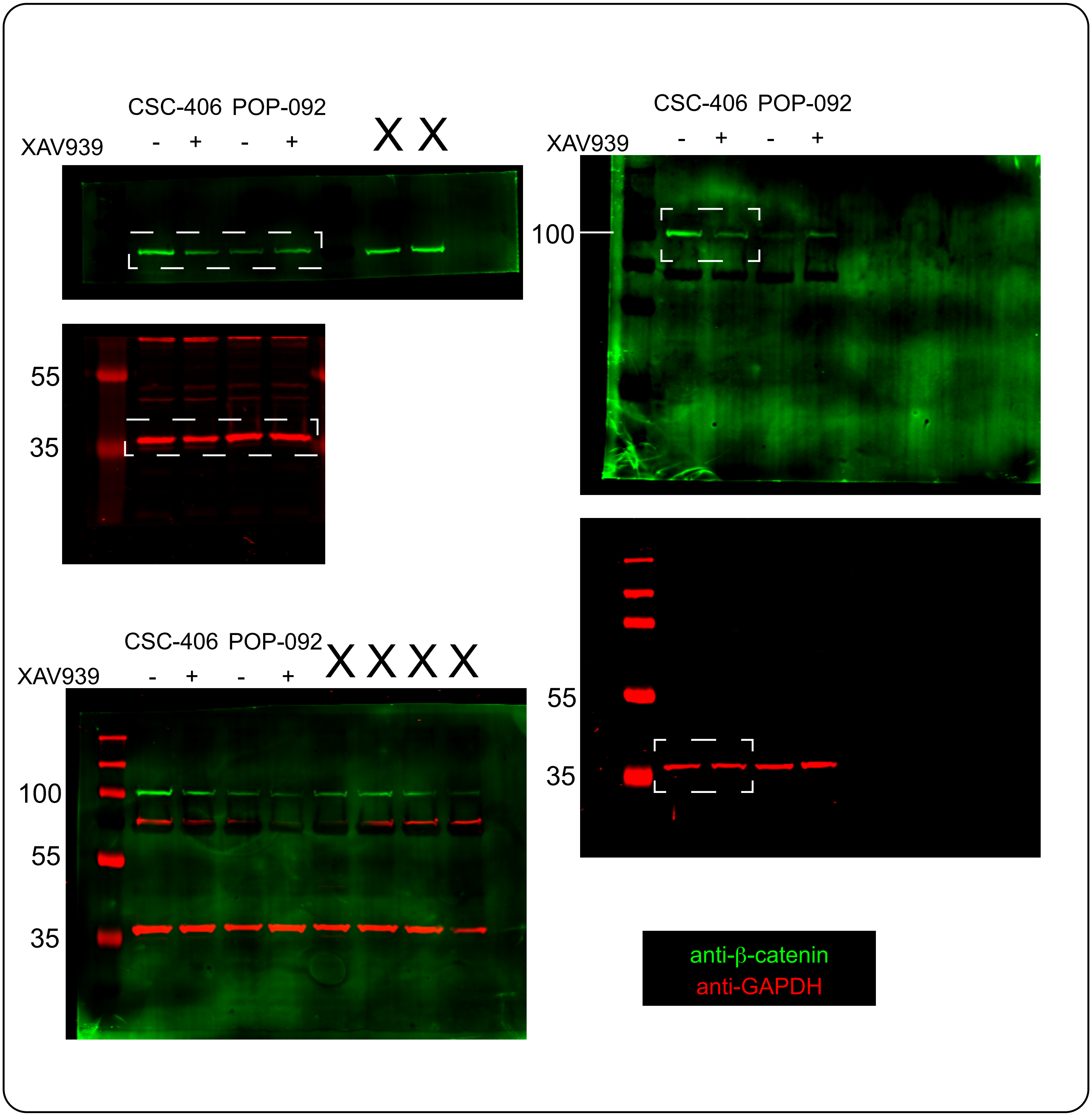

Used for Panels 3E and 4C.
Boxed regions used for figures.

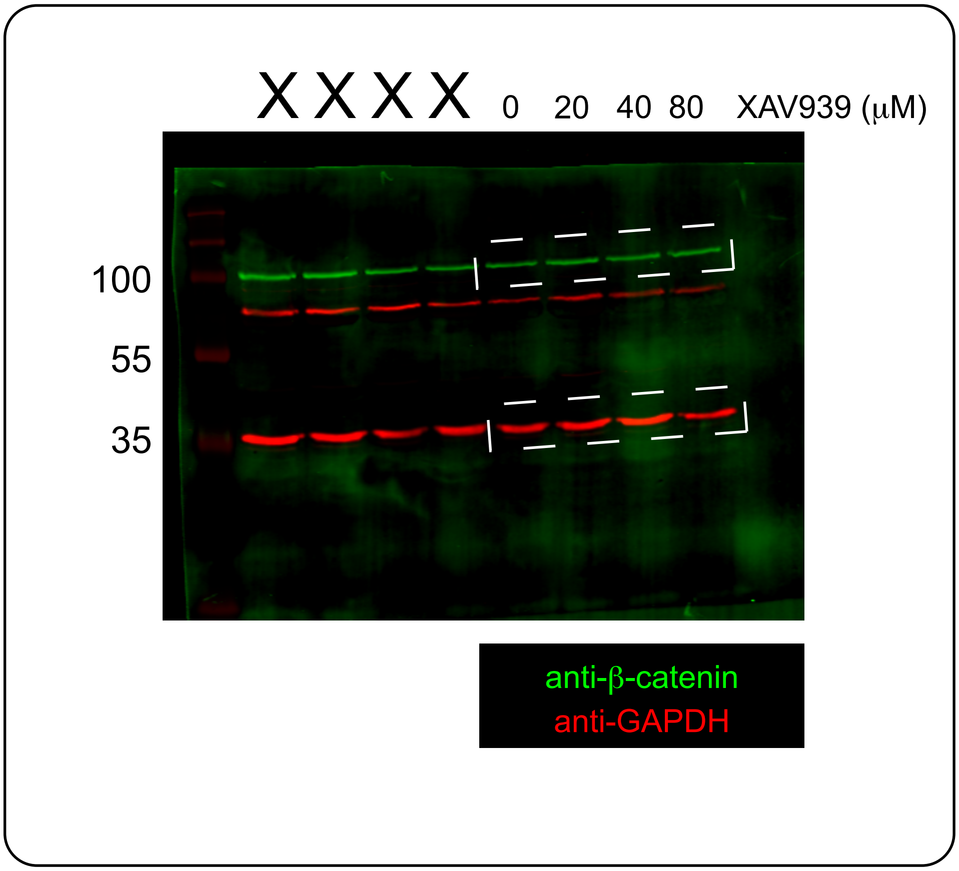

Used for Panel 4E.
Boxed regions used for figures.
